# Effects of Adrenergic Agonists and Antagonists on Cardiopulmonary Function During Normobaric Hypoxia in Rat

**DOI:** 10.3389/fphys.2019.00860

**Published:** 2019-07-05

**Authors:** Christian Bölter, Philipp Gabriel, Peter Appelt, Aida Salameh, Katrin Schierle, Beate Rassler

**Affiliations:** ^1^Carl-Ludwig-Institute for Physiology, University of Leipzig, Leipzig, Germany; ^2^Department of Pediatric Cardiology, Heart Centre, University of Leipzig, Leipzig, Germany; ^3^Institute of Pathology, University of Leipzig, Leipzig, Germany

**Keywords:** adrenergic blockade, cardiac function, norepinephrine, normobaric hypoxia, pulmonary edema

## Abstract

Pulmonary edema (PE) is an issue widely noted in acute exposure to hypoxia as seen in high altitude climbers, yet the etiology of this is not defined. Previous studies in rats showed that both hypoxia and strong sympathetic activation may induce PE. As acute exposure to hypoxia is accompanied by sympathetic activation, we assume that this may impair pulmonary circulation and contribute to the development of hypoxic PE. The aim of the present study was to investigate the effects of adrenergic agonists and antagonists as models for overstimulation and suppression, respectively, of sympathetic activity on cardiovascular function and formation of PE in hypoxic rats. Norepinephrine or adrenergic blockers were infused to rats exposed to normobaric hypoxia with 10% O_2_ over time intervals up to 24 h. Normoxic and hypoxic controls received 0.9% NaCl infusion. We evaluated hemodynamic function and lung histology. A significant decrease of left ventricular systolic function was observed after 6 h of hypoxia. This effect was less pronounced with α-adrenergic blockade but was more severe with combined α-plus β-adrenergic blockade. Norepinephrine delayed the onset of hypoxic left ventricular depression but did not reduce its degree. Significant PE developed after 16 h of hypoxia. It regressed under α- but not with β-adrenergic blockade, and was aggravated by combining hypoxia with norepinephrine. Almost half of the animals exposed to hypoxia over 16–24 h suffered cardiorespiratory arrest during the experiment and presented with signs of acute right ventricular failure. They had significantly elevated serum catecholamine concentrations and significantly stronger PE than the others. Notably, most of them had received norepinephrine or combined adrenergic blockade. Mild changes in serum catecholamine concentrations indicated that hypoxic sympathoadrenergic activation was only weak. Hence, it was not sufficient to prevent left ventricular depression. However, the results show that α-adrenergic mechanisms contribute to the formation of hypoxic PE. Adrenergic blockade but also sympathetic overactivity may induce pulmonary congestion, PE and acute right ventricular failure indicating that a fine balance of sympathetic activation under hypoxic conditions is crucial. This has important implications for climbers to high altitude as well as for patients suffering from hypoxia.

## Introduction

Acute exposure to hypoxia may induce development of pulmonary edema (PE) as can often be observed after rapid ascents to high altitude (Bärtsch et al., 2005; Korzeniewski et al., 2015). High-altitude pulmonary edema (HAPE) is initiated by an increase in pulmonary capillary pressure and is therefore considered a hydrostatic-type PE. Elevated pulmonary capillary pressure results from hypoxic pulmonary vasoconstriction that not only occurs in arterial vessels but also has a considerable venous component (Maggiorini et al., 2001). Pulmonary venoconstriction may significantly increase pulmonary capillary pressure and thus, promote edema formation. In a number of species including the human, pulmonary veins are more sensitive to vasoconstrictor stimuli than pulmonary arteries (Gao and Raj, 2005). In particular, a significantly greater vasoconstrictor effect of hypoxia was found in rat pulmonary veins than in arteries (Zhao et al., 1993).

Further, hypoxia is accompanied by activation of the sympathetic nervous system via peripheral chemoreceptor stimulation (Heistad and Abboud, 1980; Eckberg et al., 1982; Lesske et al., 1997). Numerous studies in animals and humans evidenced sympathetic activation in hypoxia by increased sympathetic nerve activity or plasma catecholamine concentrations (Johnson et al., 1983; Leuenberger et al., 1991; Morgan et al., 1995; Duplain et al., 1999; Hansen and Sander, 2003) or by abolishing sympathetic effects with adrenergic blockers under hypoxic conditions (Marshall and Metcalfe, 1988; Mazzeo and Reeves, 2003; Bates et al., 2017). This sympathetic activation is reflected in tachycardia and enhanced myocardial contractility while stroke volume remained unchanged. As a consequence, cardiac output increased under hypoxic conditions (Talbot et al., 2005; Bärtsch and Gibbs, 2007; Yan et al., 2007). The improvement of cardiac output is supported by reduction of peripheral resistance due to the vasodilator effect of tissue hypoxia. Sympathetic activation is thought to compensate for this hypoxic vasodilation of systemic arterioles and the resulting decrease in systemic blood pressure (Marshall and Metcalfe, 1988). However, strong sympathetic activation may induce formation of PE as it occurs in neurogenic PE (Busl and Bleck, 2015; Šedý et al., 2015) or as a complication of pheochromocytoma (Riester et al., 2015; Velegraki et al., 2018). Experimental studies in rats with adrenergic stimulation showed that PE particularly results from application of α-adrenergic agonists (Chen et al., 1976; Dai et al., 1993; Rassler et al., 2001). β-Adrenergic agonists may also induce PE but the edema developed more gradually and was less severe than with α-adrenergic treatment (Rassler et al., 2003). In a previous study in rats with normobaric hypoxia, we observed that hypoxia-induced PE developed in a similar time-frame and at similar severity as with infusion of norepinephrine (NE) in normoxic conditions (Rassler et al., 2007).

The question arises whether hypoxia-induced sympathetic activation may impair pulmonary circulation and contribute to the development of hypoxic PE. Notably, pulmonary veins showed a stronger response than pulmonary arteries both to vasoconstrictor effects of α_1_-adrenergic agonists and to the vasodilator effects of β_2_-adrenergic agonists (Rieg et al., 2011). The aim of the present study was to investigate the effects of adrenergic agonists and antagonists on cardiovascular function and the development of PE in rats exposed to normobaric hypoxia. Adrenergic blockade was used to assess the role of hypoxic sympathetic activation in the cardiovascular and pulmonary responses to acute hypoxia. Infusion of NE served as a model of sympathetic overactivation. This may implicate important conclusions for hypoxemic patients treated with adrenergic or antiadrenergic drugs as well as for tourists ascending to high altitude.

## Materials and Methods

### Animal Model

All experiments were performed on 216 female Sprague-Dawley rats supplied by Charles River (Sulzfeld, Germany). The body weight was 195–265 g at the beginning of the study corresponding to an age of about 12 weeks. All animal protocols were approved by the Federal State Agency. The experiments were conducted in accordance with the Guide for the Care and Use of Laboratory Animals published by the National Institutes of Health and with the “European Convention for the Protection of Vertebrate Animals used for Experimental and other Scientific Purposes” (Council of Europe, 1986).

### Study Protocol

Animals were exposed to normoxia (N) or normobaric hypoxia (H) for 1.5, 6, 16, and 24 h. For exposure to hypoxia, the animals were placed into a hypoxic chamber sized 65 cm × 105 cm × 50 cm. The gas mixture in the chamber contained 10% oxygen in nitrogen. A special equipment prevented penetration of ambient air during manipulations on the animals, thus keeping the oxygen concentration in the chamber stable at 10 ± 0.5%. Normoxic animals remained under room air condition. Additionally, all animals received an intravenous infusion over the total experimental time. Normoxic (NaCl,N) and hypoxic controls (NaCl,H) were infused with 0.9% sodium chloride solution. Norepinephrine (NE) was administered both to normoxic (NE,N) and hypoxic animals (NE,H) to simulate a strong adrenergic activation. The effects of specific (α- or β-) or combined α-plus β-adrenergic blockade were investigated under hypoxic conditions by infusion with the α-adrenergic blocker prazosin (PZ,H), the β-adrenergic blocker propranolol (PR,H) or both (PZ+PR,H; Table 1). The drug doses applied were chosen according to previous experiments (Rassler et al., 2003). Except for PZ (Pfizer, Karlsruhe, Germany), all drugs were obtained from Sigma-Aldrich (Deisenhofen, Germany).

**TABLE 1:** List of treatments.

**Group/Subgroup**	**n: total (1.5, 6, 16, 24 h)**	**Environment**	**Infusion drug and dose**
**Control**			Sodium chloride
NaCl,N	34 (9, 9, 8, 8)	Normoxia	0.9% NaCl
NaCl,H	28 (8, 7, 8, 6)	Hypoxia (10% O_2_)	0.9% NaCl
**Adrenergic blockade**			
PZ,H	25 (7, 5, 7, 6)	Hypoxia (10% O_2_)	Prazosin (PZ) 0.1 mg kg^–1^ h^–1^
PR,H	32 (5, 7, 9, 11)	Hypoxia (10% O_2_)	Propranolol (PR) 1 mg kg^–1^ h^–1^
PZ+PR,H	33 (6, 5, 11, 12)	Hypoxia (10% O_2_)	Prazosin + Propranolol PZ: 0.1 mg kg^–1^ h^–1^, PR: 1 mg kg^–1^ h^–1^
**Adrenergic stimulation**			Norepinephrine
NE,N	28 (6, 6, 8, 8)	Normoxia	NE 0.1 mg kg^–1^ h^–1^
NE,H	37 (10, 7, 10, 10)	Hypoxia (10% O_2_)	NE 0.1 mg kg^–1^ h^–1^

*n, number of animals.*

Infusions were administered with automatic pumps (Infors AG, Basel, Switzerland) at a rate of 4 ml kg^–1^ h^–1^ via an infusion catheter (Vygon, Aachen, Germany). The infusion catheter was inserted into the left jugular vein [for a more detailed description of this procedure see our previous publication (Rassler et al., 2001)]. In animals designed for infusions over 6, 16, or 24 h, this operation was performed in 2% isofluran anesthesia. These animals woke up after catheter insertion and moved freely with access to tap water and rat chow diet (Altromin C100, Altromin GmbH, Lage, Germany). Animals allocated to 1.5 h of infusion were anesthetized with an intraperitoneal (i.p.) injection of thiopental sodium (Trapanal®, Byk Gulden, Konstanz, Germany) 80 mg kg^–1^ and remained in anesthesia until the end of the experiment. Exposure to hypoxic environment started immediately after catheter insertion.

### Abnormalities in the Experimental Course and Clinical Assessment

All abnormalities in the experimental course were noted down, in particular, problems with heart catheterization and attacks of cardiorespiratory arrest. In addition, during hemodynamic measurements and at inspection of the abdominal and chest cavities, we logged the presence of tissue edema in the neck region and the appearance of liver, lung, heart and large vessels (aorta and vena cava). The most important features were size and color of the liver, color of the lung, size of the cardiac ventricles and congestion in the inferior vena cava, which were indicative of acute right ventricular failure.

### Hemodynamic Measurements

About 30–40 min before the end of the exposure time, the animals were anesthetized with thiopental (Trapanal® 80 mg kg^–1^, i.p.). They were tracheotomized, and a polyethylene cannula was placed in the trachea. The right ventricle (RV) and left ventricle (LV) were catheterized with Millar® (Millar Instruments, Houston, TX) ultraminiature catheter pressure transducers [for more details see our previous publication (Rassler et al., 2001)] to measure heart rate as well as RV and LV systolic pressures. In addition, RV and LV maximal velocities of rise (dP/dtmax) and drop in systolic pressure (dP/dtmin) were determined as measures of ventricular contractility and relaxation, respectively. After withdrawal of the LV catheter tip into the aorta, diastolic aortic pressure was measured to calculate mean aortic pressure. Cardiac index (body mass-related cardiac output) was determined by thermodilution using a thermosensitive 1.5F microprobe and a Cardiomax II computer (Columbus Instruments, Columbus, OH). The total peripheral resistance was calculated by dividing mean aortic pressure by cardiac index. Hypoxic animals remained in hypoxia until completion of hemodynamic measurements.

### Sampling of Materials

After the hemodynamic measurements, the abdominal cavity was opened by midline incision. Animals were sacrificed by drawing blood from the abdominal aorta. In the next step, we opened the thoracic wall and collected pleural fluid. After ligation of the right main bronchus, a bronchoalveolar lavage (BAL) was performed two times consecutively with 3 ml 0.9% NaCl each. The fluid was instilled via the tracheal cannula into the left lung and withdrawn immediately. The recovery rate was about 90% on average. From the intact right lung, tissue samples of the upper and lower lobes were fixated in formalin for histological analysis. A piece of the middle lobe was taken for determination of wet-to-dry (W/D) weight ratio. The right lateral lobe of the liver was excised and also fixated in formalin for histological examination. The blood was centrifuged for 10 min at 2100 rpm. Serum, pleural fluid and recovered BAL fluid were frozen and stored at −80°C for further analyses.

### Histology of Lung and Liver, BAL Cytology

The formalin-fixated tissue samples of the lung and liver were embedded in paraffin, sliced and stained with hematoxylin-eosin. Histological assessments were done by two independent investigators (PG and KS), who were blinded toward the treatment group. In the lungs, they evaluated PE, inflammation and vascular hypertrophy. For a detailed quantification of PE, the complete histological section of a lung was assessed. First, PE severity in each area of the section was gauged visually by evaluating the width of alveolar septa and the definition of alveolar spaces. PE scores ranged from 0 (absent), 1 (mild: alveolar septa slightly thickened, alveolar space well defined), 2 (moderate: thickness of alveolar septa about double the normal width, alveolar space narrowed but still defined), and 3 (severe: alveolar spaces hardly determinable and/or alveolar edema). The PE index was calculated by cumulating the products of PE score and proportionate area of each part of the histological preparation. The liver preparations were assessed for congestion.

BAL fluid was centrifuged for 20 min at 1500 rpm in a cytocentrifuge. The cytologic preparation was stained with hematoxylin-eosin and evaluated by a pathologist (KS) who was not aware of the treatment of the animal. The number of macrophages, neutrophils, lymphocytes, and eosinophils were given in per cent of the total cell number.

### Lung Wet-to-Dry Weight Ratio

Lung tissue samples were weighed immediately after preparation (wet weight, W) and after drying in an oven at 75°C for 48 h (dry weight, D). The W/D ratio served as a surrogate parameter of water accumulation in the lung.

### Catecholamine Concentration in Serum

Serum concentrations of epinephrine (Epi) and norepinephrine (NE) were measured by high-pressure liquid chromatography (HPLC) using a commercial HPLC assay (Chromsystems, Martinsried, Germany) based on the method of Bauersfeld et al. (1986). Sample processing and performance of the HPLC were carried out according to the manufacturer’s instructions. Catecholamines were detected using an electrochemical detector EC3000 (Recipe, Munich, Germany).

### Statistical Analysis

Statistical analyses were carried out with the software package SigmaPlot Version 13.0 (Systat Software GmbH, Erkrath, Germany) for Windows. All experimental groups (all treatments, all intervals of time) were statistically compared using a One Way Analysis of Variance (ANOVA) with a *post hoc* test according to the Holm–Sidak method. If the data were not normally distributed, a Kruskal–Wallis ANOVA on ranks with a *post hoc* test according to Dunn’s method was applied. Comparisons between animals with and without abnormalities were performed using a Mann–Whitney U statistics. In the text, normally distributed data are given as means ± SEM, the others are expressed as medians (25%; 75%). *P*-values <0.05 were considered significant.

## Results

### Hemodynamic Function

**FIGURE 1:**
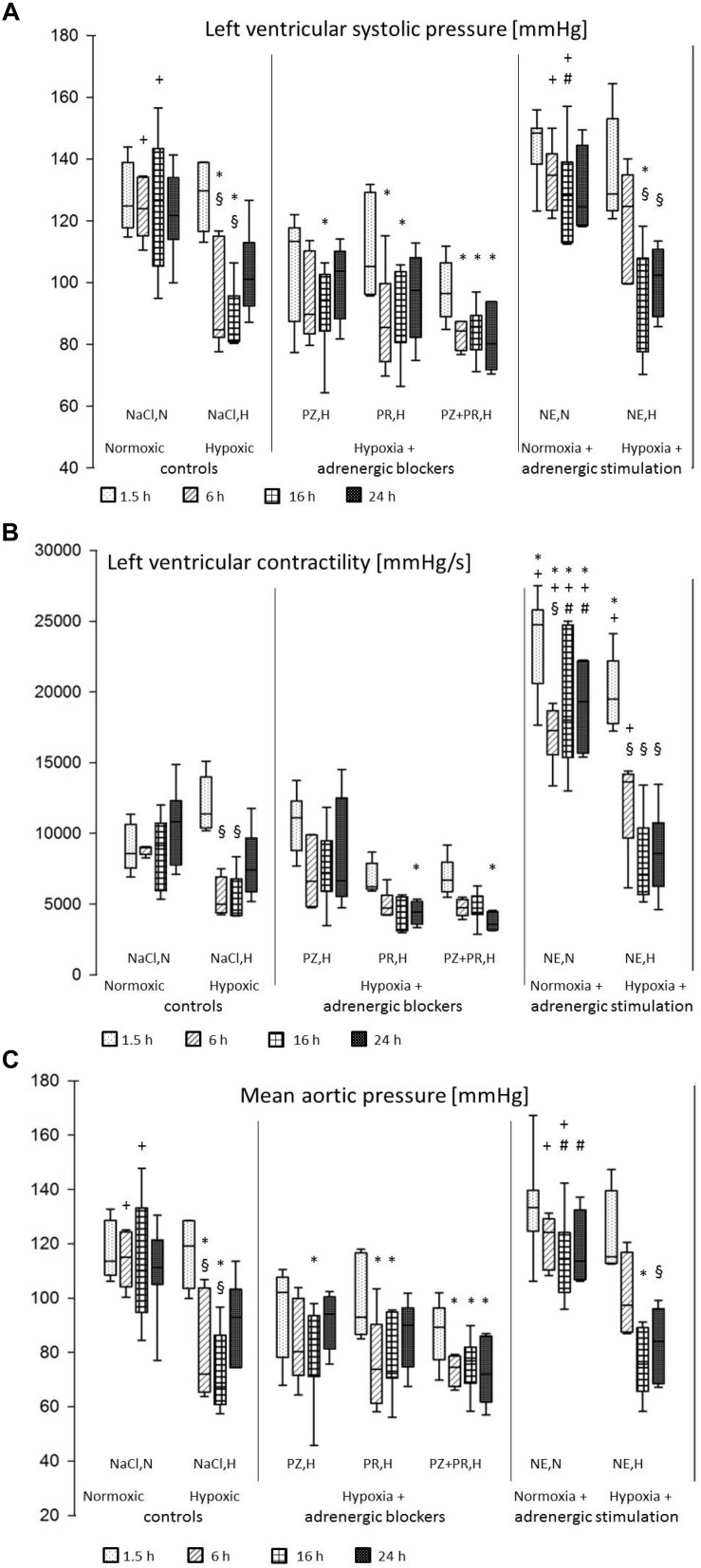
**(A)** Left ventricular systolic pressure [mmHg]; **(B)** Left ventricular contractility (LV dP/dtmax) [mmHg/s]; **(C)** Mean aortic pressure [mmHg]. For abbreviations of treatment groups see Table 1. Data are given as medians (black lines), 25th/75th percentiles (boxes), and 10th/90th percentiles (whiskers). Significance marks: ^*^significant vs. respective NaCl,N; ^+^significant vs. respective NaCl,H; ^#^significant vs. respective NE,H; ^§^ significant vs. 1.5 h of same treatment.

The results of the hemodynamic measurements are presented in Figures 1A–C and in Table 2.

**TABLE 2:** Effects of hypoxia and/or adrenergic and antiadrenergic treatment on hemodynamic function.

	**NaCl,N**	**NaCl,H**	**PZ,H**	**PR,H**	**PZ+PR,H**	**NE,N**	**NE,H**
**LV dP/dtmin [mmHg/s]**							
1.5 h	−12945 (−17662; −11799)	−9577 (−17305; −7261)	−7303 (−11736; −5246)	−6615 (−10191; −6355)	−5660 (−8401; −4145)	−10634 (−11862; −9587)	−11372 (−14044; −10234)
6 h	−11707 (−12191; −7749)	−6816 (−10031; −4186)	−7586 (−13299; −4788)	−4720 (−8278; −4279)	−3264 (−6115; −3155)	−9550 (−10253; −8300)	−7668 (−12754; −6353)
16 h	−9612 (−12250; −6950)	−4618 (−8337; −4058)	−8809 (−11257; −5458)	−4711 (−5302; −4245)	−4334 (−5893; −3337)	−10000 (−12330; −7797)	−5729 (−8267; −5141)
24 h	−9549 (−11946; −6948)	−8979 (−12066; −6261)	−7439 (−11753; −5882)	−5098 (−6706; −3642)	−3625 (−4268; −2808)	−9884 (−10006; −9712)	−7071 (−8281; −5650)
**DAP [mmHg]**							
1.5 h	102.4 (99.2; 118.2)	103.2 (94.2; 118.0)	91.0 (68.9; 97.5)	80.7 (76.9; 104.1)	80.6 (65.5; 87.1)	116.7 (110.0; 126.4)	109.5 (98.2; 125.9)
6 h	106.0 (93.1; 114.5)^+^	61.2 (48.7; 92.4) ^*^,^§^	73.3 (58.3; 89.6)	62.0 (48.1; 80.8)^*^	64.8 (56.8; 69.8)^*^	108.3 (97.2; 113.7)^+^	86.9 (76.1; 114.9)
16 h	91.6 (83.9; 126.1)^+^	51.3 (40.6; 76.9)^*^,^§^	75.7 (57.2; 84.3)	64.1 (59.9; 85.4)	68.2 (59.0; 74.4)^*^	95.7 (87.5; 110.6)^+,#^	61.4 (52.7; 70.2)^*^,^§^
24 h	100.7 (91.2; 108.9)	84.8 (59.7; 93.5)	84.6 (74.3; 90.7)	79.3 (67.1; 88.4)	63.7 (50.7; 78.2)^*^	102.2 (95.0; 120.4)^#^	61.4 (48.1; 82.8)^*^,^§^
**RVSP [mmHg**]							
1.5 h	18.6 (15.2; 23.0)	22.7 (16.3; 28.2)	27.7 (22.3; 34.4)	26.8 (26.6; 32.8)	25.2 (18.1; 27.9)	38.5 (33.3; 55.4)^*^	27.2 (23.8; 31.3)
6 h	18.3 (16.7; 22.4)	26.8 (26.0; 31.0)	23.2 (23.0; 33.9)	25.4 (20.9; 30.4)	20.9 (18.4; 23.4)	70.8 (57.6; 86.8)^*^	26.6 (25.0; 29.9)
16 h	25.7 (18.6; 32.1)	25.7 (20.6; 28.8)	22.5 (20.8; 25.3)	20.9 (17.7; 22.4)	24.1 (18.5; 28.2)	61.9 (48.9; 72.3)	29.0 (23.7; 35.8)
24 h	23.2 (19.7; 25.6)	26.4 (21.7; 32.5)	23.7 (22.8; 33.7)	21.1 (16.3; 30.4)	21.1 (19.2; 23.8)	53.9 (38.6; 56.1)^*^	29.8 (26.7; 34.0)
**RV dP/dtmax [mmHg/s]**							
1.5 h	1107 (787; 1266)	1203 (845; 2164)	1613 (1261; 3109)	1525 (1323; 1860)	1376 (1251; 1838)	4355 (2547; 6653)^*^,^+^	1348 (1147; 1964)
6 h	1231 (1000; 1496)	1480 (1253; 1681)	1840 (1463; 2497)	1451 (982; 1612)	1193 (884; 1339)	4120 (2844; 7750)	1548 (1343; 2669)
16 h	1766 (1179; 2625)	1760 (1434; 2208)	1521 (1361; 2471)	1154 (977; 1334)	1187 (867; 1354)	5100 (4155; 6136)	2035 (1593; 2848)
24 h	1298 (1025; 1409)	2316 (1449; 2769)	1593 (1372; 3216)	963 (867; 1383)	1148 (1000; 1237)	3605 (2647; 4436)	2227 (1853; 3459)
**RV dP/dtmin [mmHg/s]**							
1.5 h	−1324 (−1597; −944)	−1131 (−1806; −728)	−1771 (−2256; −1254)	−1561 (−2569; −1196)	−1439 (−1752; −724)	−2589 (−4136; −1880)	−2366 (−4136; −1414)
6 h	−1580 (−1935; −892)	−1343 (−1649; −1135)	−1518 (−2150; −1215)	−1627 (−2357; −940)	−728 (−1354; −691)	−4000 (−4875; −2819)^+^	−1393 (−2499; −1108)
16 h	−1584 (−2475; −1119)	−1549 (−1991; −909)	−1304 (−1796; −1035)	−918 (−1071; −672)	−915 (−1231; −765)	−3718 (−6063; −3386)	−1762 (−2261; −1221)
24 h	−1425 (−2153; −959)	−1433 (−2121; −1146)	−2053 (−2946; −1131)	−801 (−1648; −557)	−893 (−1208; −849)	−2718 (−3404; −2261)	−2212 (−2506; −1724)
**HR [min^–1^]**							
1.5 h	428 (411; 456)	400 (382; 418)	384 (376; 416)	296 (266; 337)	283 (253; 331)	491 (470; 510)^+^	422 (378; 459)
6 h	461 (416; 489)	368 (330; 414)	357 (336; 386)	314 (293; 331)	267 (226; 288)^*^	475 (467; 505)^+^	419 (325; 445)
16 h	419 (404; 451)	398 (350; 441)	379 (327; 417)	312 (290; 364)	293 (250; 328)	522 (463; 554)^+^	440 (383; 475)
24 h	466 (430; 492)	420 (373; 439)	436 (371; 452)	312 (283; 374)^*^	298 (278; 329)^*^	523 (494; 542)	470 (446; 475)
CI [ml^*^min^–1*^kg^–1^]							
1.5 h	272 (224; 347)	383 (300; 477)	316 (216; 408)	326 (285; 337)	319 (266; 357)	320 (239; 416)	414 (322; 449)
6 h	348 (228; 392)	350 (283; 418)	374 (305; 440)	329 (269; 402)	298 (256; 317)	184 (163; 259)	259 (194; 466)
16 h	233 (225; 236)	344 (252; 364)	475 (443; 512)^*^°	282 (253; 308)	281 (185; 340)	317 (226; 340)	255 (231; 315)
24 h	372 (260; 499)	284 (266; 317)	333 (236; 416)	269 (238; 302)	241 (202; 306)	295 (257; 378)	200 (179; 323)
TPR [mmHg^*^min^*^kg^*^ml^–1^]							
1.5 h	0.37 (0.35; 0.48)	0.30 (0.25; 0.40)	0.28 (0.25; 0.44)	0.34 (0.27; 0.37)	0.28 (0.24; 0.34)	0.42 (0.32; 0.53)	0.27 (0.25; 0.46)
6 h	0.31 (0.27; 0.53)	0.28 (0.17; 0.31)	0.24 (0.18; 0.28)	0.27 (0.16; 0.33)	0.23 (0.22; 0.31)	0.57 (0.46; 0.70)^+^	0.28 (0.22; 0.69)
16 h	0.54 (0.40; 0.59)	0.19 (0.17; 0.37)	0.18 (0.17; 0.20)^*^	0.31 (0.24; 0.33)	0.27 (0.24; 0.42)	0.38 (0.33; 0.48)	0.30 (0.24; 0.34)
24 h	0.33 (0.20; 0.38)	0.32 (0.30; 0.36)	0.26 (0.24; 0.37)	0.32 (0.31; 0.37)	0.28 (0.26; 0.37)	0.40 (0.36; 0.42)	0.46 (0.24; 0.49)

Data are medians (25%; 75%). LV dP/dtmin, left ventricular relaxation velocity; DAP, diastolic aortic pressure; RVSP, right ventricular systolic pressure; RV dP/dtmax, right ventricular contractility; RV dP/dtmin, right ventricular relaxation velocity; HR, heart rate; CI, cardiac index; TPR, total peripheral resistance. Abbreviation of treatments see Table 1. Significance marks: ^*^sign. vs. respective NaCl,N; ^+^sign. vs. respective NaCl,H; ^#^sign. vs. respective NE,H; °sign. vs. respective PR,H; ^§^ sign. change vs. 1.5 h of treatment.

#### Effects of Hypoxia

Hypoxia induced a depression of left ventricular function. While LV systolic pressure was still at normoxic level after 1.5 h of hypoxia (128 ± 4 mmHg), it decreased significantly with prolonged hypoxic exposure by more than 35 mmHg (*p* < 0.01). A tendency of recovery occurred after 24 h of hypoxia (103 ± 6 mmHg; Figure 1A). Contractility of the LV (LV dP/dtmax) was also significantly reduced after 6–16 h of hypoxia to about 50% of normoxic values (Figure 1B). LV dP/dtmin decreased to a similar range but this was not significant. In contrast, RV systolic pressure as well as dP/dtmax and dP/dtmin remained constant under hypoxic conditions. An insignificant decrease in heart rate and increase in cardiac index was noted in early stages of hypoxia, both of which recovered with longer exposure to hypoxia. Hypoxia did not induce a significant change in total peripheral resistance but diastolic aortic pressure decreased significantly. As a consequence of the depression of LV systolic function and the decrease in diastolic aortic pressure, mean aortic pressure was also significantly reduced in hypoxia (Table 2 and Figure 1C).

#### Hypoxia and Adrenergic Blockers

Combination of hypoxia with adrenergic blockade did not substantially improve LV systolic function. While the α-blocker PZ slightly attenuated the hypoxic depression of LV function and aortic pressures, β-blockade with PR had no effect at all. Blockade of both α- and β-adrenoceptors even deteriorated the situation of the animals as this treatment abolished the recovery of LV systolic pressure after 24 h (81 ± 4 mmHg; Figure 1A). RV systolic pressure, dP/dtmax and dP/dtmin remained unchanged compared to hypoxic controls. Hypoxia combined with PZ significantly reduced total peripheral resistance after 16 h of hypoxia (0.18 [0.17; 0.20] mmHg min kg ml^–1^) compared to respective normoxic controls (0.54 [0.40; 0.59]; *p* = 0.002). Consequently, cardiac index increased significantly under this condition (475 [443; 512] ml min^–1^ kg^–1^) compared to respective normoxic controls (233 [225; 236]; *p* < 0.001), while PR or PZ+PR rather decreased cardiac index (Table 2).

#### Effects of NE in Normoxia and Hypoxia

Short-term (1.5 h) NE infusion in normoxic animals improved LV systolic pressure slightly but not significantly compared to respective normoxic controls. This effect gradually declined with prolonged NE treatment (6 h and more). Compared to hypoxic controls, LV systolic pressure was significantly elevated after 6 and 16 h of NE infusion (134 ± 4 and 130 ± 6 mmHg, respectively; *p* < 0.001; Figure 1A), and similarly were mean and diastolic aortic pressures. NE significantly increased LV contractility to about two to threefold control values while LV dP/dtmin remained unaffected. Total peripheral resistance increased with NE infusion and was significantly higher than in hypoxic controls after 6 h (0.57 [0.46; 0.70] vs. 0.28 [0.17; 0.31] mmHg min kg ml^–1^; *p* = 0.009) but then returned to control levels. RV function was considerably improved by NE infusion in normoxia (38 [33; 55] mmHg) compared to respective normoxic controls (19 [15; 23]; *p* = 0.02) but in hypoxia, it immediately decreased to control level (27 [24; 31] mmHg; Table 2). NE infusion did not alter hypoxia-induced changes in LV systolic pressure, mean aortic pressure (Figures 1A,C), heart rate and cardiac index (Table 2). Only LV dP/dtmax remained significantly elevated for the first 6 h of hypoxic exposure (Figure 1B).

### Pulmonary Edema and Pleural Fluid

**FIGURE 2:**
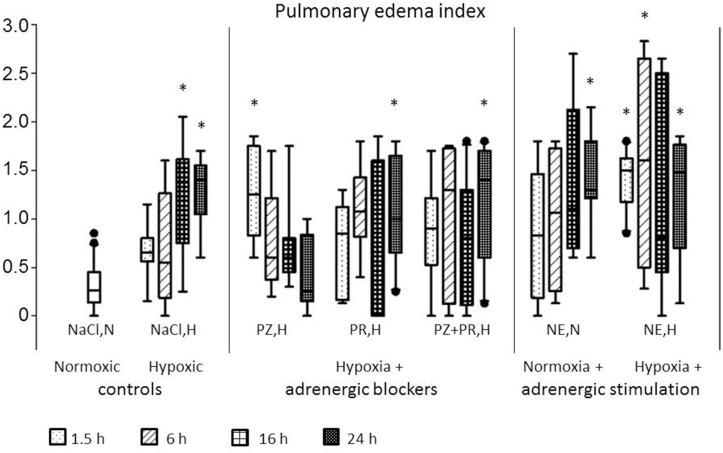
Pulmonary edema index. For abbreviations of treatment groups see Table 1. Normoxic controls are pooled; in the other treatment groups, the four boxes represent treatments over 1.5, 6, 16, and 24 h, respectively. Data are given as medians (black lines), 25th/75th percentiles (boxes), 10th/90th percentiles (whiskers), and outliers (circles). Significance marks: ^*^significant vs. NaCl,N.

**FIGURE 3:**
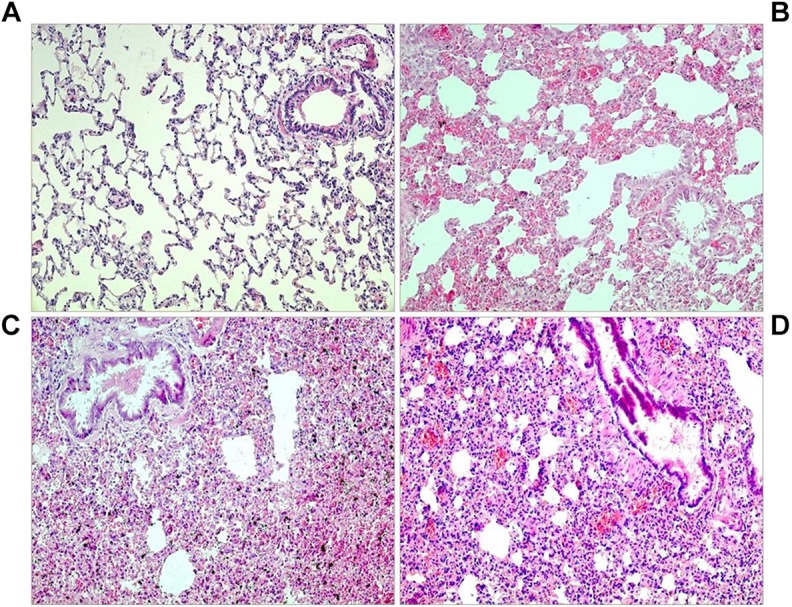
Lung histology. **(A)** Normoxic control: normal lung tissue without edema. **(B)** Twenty four hours NaCl,H: moderate interstitial edema. **(C)** Twenty four hours NE,H: severe edema. **(D)** Twenty four hours PZ+PR,H: severe edema, blood congestion (all slices: original magnification 20×).

In normoxic controls, histologic signs of PE were rare and only mild (PE index 0.26 [0.14; 0.45]). The PE index significantly increased with prolonged exposure to hypoxia (16 h NaCl,H: 1.38 [0.75; 1.61]; *p* = 0.008, and 24 h NaCl,H: 1.40 [1.05; 1.55]; *p* = 0.016). The edema occurred in patches that were spread over the total lung. With α-adrenergic blockade, PE developed very fast with a significantly elevated PE index after 1.5 h (1.25 [0.83; 1.75]; *p* = 0.007) but regressed after several hours of treatment. With β-adrenergic blockade (PR,H and PZ+PR,H), the PE index increased gradually and reached its highest value after 24 h (1.00 [0.65; 1.65]; *p* = 0.017, and 1.40 [0.60; 1.70]; *p* = 0.002, respectively). NE infusion under normoxic conditions increased the PE index significantly after 24 h of infusion (1.30 [1.21; 1.80]; *p* < 0.001), but most pronounced edema was induced by NE infusion combined with hypoxic exposure (24 h NE,H 1.48 [0.70; 1.76]; *p* = 0.005; Figures 2, 3).

PE was confined to the interstitium with alveoli remaining free from fluid in all animals. This was also confirmed by the W/D ratio that was 4.8 [4.6; 5.0] in normoxic controls and ranged between 4.7 and 5.4 in all other groups (*p* = 0.28; data not shown). There were no histologic symptoms of inflammation or vascular hypertrophy in the lungs. Moreover, no significant changes in the distribution of macrophages, neutrophils and lymphocytes were found in BAL cytology (data not shown).

PE is often associated with pleural transudation. In normoxic controls, the amount of pleural fluid was 0.12 [0.05; 0.48] ml. Similar amounts were found in hypoxic control rats (0.05–0.25 ml). Adrenergic blockade over 16 to 24 h, particularly with PR,H and PZ+PR,H, increased pleural fluid to about threefold. The highest pleural fluid amounts, however, resulted from 16 to 24 h of NE,N treatment (2.90 [0.98; 4.35]; *p* = 0.008 and 2.00 [0.72; 4.38] ml; *p* = 0.041, respectively; Table 3).

**TABLE 3:** Effects of hypoxia and/or adrenergic and antiadrenergic treatment on the amount of pleural fluid.

	**NaCl,N**	**NaCl,H**	**PZ,H**	**PR,H**	**PZ+PR,H**	**NE,N**	**NE,H**
**pleural fluid [ml]**							
1.5 h		0.05 (0.0; 0.07)	0.15 (0.05; 0.25)	0.10 (0.05; 0.10)	0.05 (0.05; 0.10)	0.02 (0.0; 0.32)	0.08 (0.05; 0.27)
6 h	0.12 (0.05; 0.48)	0.20 (0.10; 1.25)	0.15 (0.10; 0.40)	0.15 (0.05; 0.20)	0.20 (0.10; 0.30)	2.10 (0.42; 3.00)	1.40 (0.90; 2.00)
16 h		0.20 (0.12; 1.19)	0.18 (0.05; 2.98)	0.40 (0.12; 1.50)	0.60 (0.12; 1.85)	2.90 (0.98; 4.35)^*^	0.65 (0.19; 3.38)
24 h		0.25 (0.05; 0.70)	0.10 (0.04; 1.30)	0.18 (0.05; 2.25)	0.40 (0.10; 3.80)	2.00 (0.72; 4.38)^*^	0.45 (0.05; 3.25)

Normoxic controls are pooled. Data are medians (25%; 75%). Abbreviation of treatments see Table 1. Significance marks: ^*^sign. vs. NaCl,N.

### Serum Concentrations of Epi and NE

In most groups, we found huge interindividual differences in serum concentrations of Epi and NE. With respect to the experimental course, we eliminated all animals presenting procedural or clinical abnormalities. These animals were dealt with separately (see below). In the remaining animals without obvious abnormalities, hypoxia caused an early increase in Epi and NE concentrations. The highest NE concentrations were found in animals with NE infusion, but all these changes were not significant (Table 4).

**TABLE 4:** Effects of hypoxia and/or adrenergic and antiadrenergic treatment on the plasma concentration of norepinephrine (NE) and epinephrine (Epi).

	**NaCl,N**	**NaCl,H**	**PZ,H**	**PR,H**	**PZ+PR,H**	**NE,N**	**NE,H**
**NE concentration [pg/ml]**							
1.5 h	870 (439; 2059)	2186 (881; 2597)	2487 (1155; 3753)	1649 (1345; 1896)	1563 (1383; 1741)	1369 (634; 4433)	4601 (1759; 28964)
6 h	1722 (1474; 3426)	2688 (1673; 3954)	1653 (780; 2577)	1254 (1047; 2102)	1662 (1017; 2616)	25092 (5702; 44482)	4884 (4543; 9936)
16 h	2510 (1130; 3891)	2340 (1799; 11165)	2219 (1009; 2871)	2355 (3104; 5515)	938 (590; 4115)	5454 (4017; 24532)	12869 (7630; 20648)
24 h	2090 (1505; 2198)	1103 (577; 1629)	5420 (2247; 6640)	3179 (2280; 7429)	1372	15747 (14634; 16859)	3935
**Epi concentration [pg/ml]**							
1.5 h	4892 (1292; 8026)	7285 (5072; 8050)	3980 (2086; 19746)	4759 (3927; 11285)	6542 (4290; 9930)	1821 (454; 4842)	9864 (4623; 11438)
6 h	3080 (2433; 5631)	10288 (6243; 14522)	4313 (2520; 4801)	5218 (2151; 5712)	8121 (3866; 9010)	14610 (1758; 27462)	4857 (2657; 7462)
16 h	5737 (4239; 7234)	4681 (3527; 16293)	4112 (2655; 5570)	6384 (3606; 7773)	3863 (653; 9916)	5522 (2386; 47340)	10438 (5866; 43598)
24 h	3993 (2557; 5288)	2257 (552; 3961)	10526 (3572; 13720)	6592 (4201; 12614)	1157	16507 (13891; 19123)	4691

*This table only contents data from animals with regular experimental course (without abnormalities). Data are medians (25%; 75%). Abbreviation of treatments see Table 1.*

### Abnormalities in the Experimental Course

**FIGURE 4:**
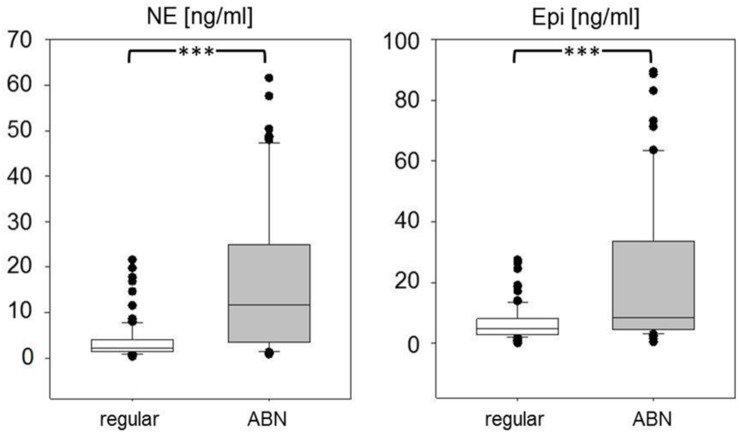
Serum concentrations of norepinephrine (NE) and epinephrine (Epi) in animals with regular experimental course (white boxes) and with clinical and/or procedural abnormalities (ABN, gray boxes). Data are given as medians, 25th/75th percentiles (boxes), 10th/90th percentiles (whiskers), and outliers (circles). Significance marks: ^∗∗∗^*p* < 0.001.

During the experiment, we observed clinical or procedural abnormalities in 81 out of 216 animals. These abnormalities meant stressful situations for the animals as reflected in significantly increased serum concentrations of Epi to about 160% and, even more, of NE to 550% of animals with regular experimental course (*p* < 0.001; Figure 4).

In a small subgroup of animals with abnormalities (*n* = 13), insertion of the LV catheter was extremely difficult due to marked constriction of the carotid artery after incision. The others showed considerable clinical abnormalities such as attacks of cardiorespiratory arrest during thiopental narcosis requiring resuscitation (*n* = 61) and/or symptoms of acute RV failure at necropsy (*n* = 42). Of these, 90% died prematurely. This group included 51% of all NE,H animals and 41% of all animals with any type of adrenergic blockade. Almost two thirds of them (*n* = 43, 63%) were exposed to hypoxia for 16 or 24 h. None of the normoxic control rats was involved in this group.

**FIGURE 5:**
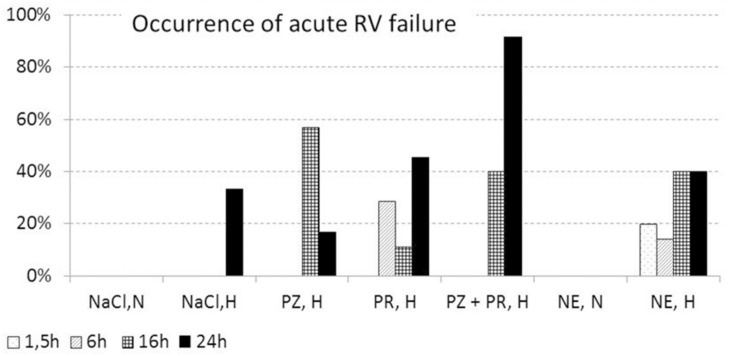
Percentage of animals with signs of acute right ventricular failure (related to total number of animals per group). For explanation of treatment groups see Table 1.

Symptoms indicating acute RV failure were a dilated right heart, a red and blotchy lung with signs of congestion (see Figure 3D), a huge dark violet liver with histological signs of congestion, and tissue edema in the neck region. They were found in 42 animals. These animals had a significantly higher PE index (1.35 [0.60; 1.70]) than rats without abnormalities (0.80 [0.30; 1.55], *p* = 0.048). The majority of them (*n* = 36, 86%) were exposed to hypoxia over 16 or 24 h, among them 11 out of 12 rats with PZ+PR infusion over 24 h (Figure 5). Thirty-five rats in this subgroup additionally suffered attacks of cardiorespiratory arrest.

## Discussion

The main results of this study were: (a) Hypoxia induced a depression in LV systolic function that was deteriorated with β-adrenergic or, even more, with combined α- and β-adrenergic blockade and was not fully compensated with NE administration; (b) Hypoxia induced an interstitial PE that was slightly attenuated with α-adrenergic blockade and increased with NE application; (c) Under prolonged hypoxia exposure, both combined adrenergic blockade and NE application may induce acute RV failure. These results may have great importance for hypoxemic patients being treated with adrenergic or antiadrenergic drugs. They have also implications for tourists to high altitude who are not well-acclimatized and overactivate their sympathoadrenal system by doing too much of effort.

It is well established that acute exposure to hypoxia activates the sympathetic nervous system by peripheral chemoreceptor reflexes (Heistad and Abboud, 1980; Eckberg et al., 1982; Lesske et al., 1997). Sympathetic activation leads to an increase in heart rate and in cardiac output (Talbot et al., 2005; Bärtsch and Gibbs, 2007; Yan et al., 2007). In humans, an increase in sympathetic nerve activity occurs within 30 min of moderate hypoxia (Leuenberger et al., 1991). These effects contribute, at least in parts, to the maintenance of LV systolic pressure (Phillips et al., 1988; Cargill et al., 1995). Echocardiographic studies in humans demonstrated that acute or subacute exposure to hypoxia even increased LV contractile function (Huez et al., 2005; Goebel et al., 2013; Rao et al., 2015). In contrast to these findings in humans, we observed an overt depression in LV systolic function in rats under hypoxic conditions. After 6 h of hypoxia, LV and aortic pressures decreased significantly. Moreover, cardiac index and heart rate remained at the level of normoxic controls indicating that sympathetic activation was absent or weak. Weak sympathoadrenergic stimulation is suggested by slight but insignificant changes in serum NE and Epi concentrations under hypoxic conditions. A study on sympathoadrenal responses to hypoxia in rats showed that the sympathetic nervous system was not stimulated during the first 12 h of exposure at 10.5% oxygen but adrenal medullary activity was enhanced (Johnson et al., 1983). However, hypoxia also increases NE clearance (Leuenberger et al., 1991), which might attenuate effects of adrenergic stimulation. Hypoxic sympathoadrenergic activation was not sufficient to maintain the LV systolic function over several hours of hypoxia, but LV function was even more decreased after β-adrenergic blockade. This was most pronounced when both α- and β-adrenergic blockers were administered. With this treatment (PZ+PR,H), LV systolic pressure and aortic blood pressure were significantly lower than in normoxic control animals. After 24 h of hypoxia, we found a slight tendency to recovery in LV systolic function that was completely absent with combined α- and β-adrenergic blockade. These findings demonstrate the depressing effect of hypoxia on LV function and emphasize the important role of hypoxic sympathetic activation for the maintenance of the LV pump function and the systemic blood pressure.

Due to the weak sympathetic effects, hypoxic depression of LV function was not fully compensated. Even NE infusion at a dose that would be sufficient to induce LV hypertrophy after 48 h (Irlbeck et al., 1996) simulating an extremely strong sympathetic activation, could only delay but not completely prevent the decrease in systemic blood pressure. Consequently, we must assume additional factors compromising LV function under hypoxic conditions. A study in dogs with progressive normobaric hypoxia demonstrated a decrease in coronary blood flow resulting in a decrease in myocardial oxygen consumption and consequently, in a depression of LV contractility (Walley et al., 1988). Impaired LV contractility due to changes in energy metabolism in the LV has also been confirmed in other animal species such as rats, and in humans (Rumsey et al., 1999; Holloway et al., 2011). In rats, hypoxia induced a loss in fatty acid oxidation and mitochondrial respiration in the LV, which were associated with a decrease in ATP synthesis (Essop et al., 2004; Bruder and Raff, 2010; Ashmore et al., 2014). Accumulation of fatty acids, particularly of arachidonic acid, in the hypoxic myocardium correlated positively with contractile dysfunction (Burton et al., 1986; de Groot et al., 1993).

Although LV systolic function is – at least in parts – preserved under hypoxia by compensatory mechanisms such as sympathetic activation or hypoxia-induced systemic vasodilation (Yan et al., 2007; Dedobbeleer et al., 2013), many studies in animals and humans reported LV diastolic dysfunction (Itoh et al., 2009; Holloway et al., 2011; Rao et al., 2015). Pulmonary hypertension resulting from hypoxic pulmonary vasoconstriction may disturb and prolong RV ejection, thus impairing early LV filling through interventricular interaction (Itoh et al., 2009). This is in correspondence with the present observation of an impaired relaxation of the LV as reflected in a marked decrease in LV dP/dtmin after 6–16 h of hypoxia.

Hypoxic pulmonary vasoconstriction, however, is not only confined to pulmonary arteries but also occurs in pulmonary veins to a considerable extent (Zhao et al., 1993). As a consequence, pulmonary capillary pressure increases causing fluid filtration into the pulmonary interstitium and formation of PE. HAPE as the most typical hypoxia-induced PE is characterized as a non-cardiogenic edema caused by elevated pulmonary capillary pressure due to an uneven hypoxic pulmonary vasoconstriction and regional overperfusion (Swenson et al., 2002; Bärtsch et al., 2005; Hopkins et al., 2005). In addition, hypoxia impairs the transcellular electrolyte transport in alveolar epithelial cells and thus inhibits alveolar fluid reabsorption (Wodopia et al., 2000; Mairbäurl et al., 2002). In the present study, significant PE occurred after 6 h of hypoxia and progressed over time of exposure, thus confirming previous results (Rassler et al., 2007). The edema was mainly confined to the pulmonary interstitium while the alveoli largely remained free from fluid. It resembled the PE induced by NE infusion under normoxic conditions that has been investigated previously (Rassler et al., 2001, 2003). In those studies, we could demonstrate that both α- and β-adrenergic mechanisms contributed to the formation of PE, but the edema was more severe with α- than with β-adrenergic stimulation. While α-adrenergic stimulation induced only minor pleural effusion but severe PE, large amounts of pleural fluid were found after β-adrenergic stimulation indicating that this fluid drainage mechanism was effective in attenuating PE formation (Rassler et al., 2003).

Addition of NE infusion to hypoxia significantly aggravated the edema suggesting that adrenergic stimulation contributes to the development of hypoxic PE. Notably, even under combined adrenergic and hypoxic stimulation, there were no signs of capillary wall destruction as indicated by the lack of alveolar edema in the histologic preparations as well as by the lung W/D ratios of less than 6.0 (Negrini et al., 1996). The α-adrenergic blocker PZ attenuated the edema while β-blockers had no significant effect indicating that particularly α-adrenergic mechanisms are involved in the formation of hypoxic PE. α-Adrenergic mediation of PE formation plays a pivotal role in several human pathologies such as neurogenic PE (Busl and Bleck, 2015; Šedý et al., 2015) or PE as a complication of pheochromocytoma (Riester et al., 2015; Velegraki et al., 2018). On the contrary, β-adrenergic mechanisms have anti-edematous and protective effects on the lungs. β_2_-Agonists decrease the permeability of the alveolo-capillary barrier and improve pulmonary fluid clearance, thus directly counteracting the effect of hypoxia and preventing edema formation (McAuley et al., 2004; Mutlu et al., 2004; Perkins et al., 2008). With β-adrenergic blockade (PR), PE deteriorated with prolonged exposure to hypoxia. In contrast, suppression of α-adrenergic effects (PZ) under hypoxia with maintenance of β-adrenergic mechanisms rapidly induced significant PE that gradually regressed over time of hypoxic exposure. The early edema in this group suggests that β-adrenergic mechanisms were not fully activated at the beginning of hypoxic exposure but with progressive activation edema formation was reduced. However, suppression of both α- and β-adrenergic effects (PZ+PR) demonstrated that a complete lack of sympathetic activation during a prolonged exposure to hypoxia might have fatal effects. After 24 h, we observed a significant depression of LVSP and a significant degree of PE. All but one of these animals presented with symptoms of acute RV failure. We assume that insufficient compensation for the hypoxic LV depression due to blockade of β-adrenergic mechanisms in combination with enhanced vasodilation due to α-adrenergic blockade promoted edema formation and induced congestion in the lungs and finally, acute right heart failure.

In contrast, the combination of hypoxia and NE application might serve as a model for sympathetic overactivation such as in situations with stress or increased physical activity after rapid ascent to high altitude. PE developed within the first 90 min of this treatment and remained at this level up to 24 h. Significant amounts of pleural transudate indicated that fluid drainage from lung tissue was activated but insufficient to prevent edema formation. The stimulating effect of NE on cardiac function decreased rapidly under hypoxic conditions, probably due to an increased NE clearance (Leuenberger et al., 1991). After 16–24 h of hypoxia, about 50% of these rats suffered cardiorespiratory arrest. Typically, attacks of cardiorespiratory arrest started after induction of anesthesia and during hemodynamic measurements indicating that hypoxia plus NE administration produce a vulnerable state that can very easily turn into decompensation. Additional stress during the first hours to days in hypoxia may promote the formation of PE and deteriorate cardiac function up to cardiorespiratory failure. This may be a serious problem for tourists rapidly ascending to high altitudes and physically overexerting themselves.

### Limitations of the Study

The present experiments were performed on female rats only to ensure comparability with previous studies (Rassler et al., 2001, 2003, 2007). One might argue that in female rats the estrous cycle may increase the variability of cardiocirculatory function. Several studies demonstrated, however, that parameters like mean arterial blood pressure and heart rate are not affected by the estrous cycle (Sharp et al., 2002; Dayton et al., 2016). In addition, an analysis of 142 heart, lung, vascular, kidney, and blood phenotypes revealed a similar variability in these traits between male and female rats (Dayton et al., 2016). Another limitation is the lack of data on the NE metabolism and excretion in the hypoxic rats. Serum NE concentrations alone do not adequately reflect the sympathoadrenergic activation as the NE clearance is also increased during acute hypoxia (Leuenberger et al., 1991).

## Conclusion

In rats, hypoxia induced a weak sympathetic activation that was not sufficient to prevent a depression of LV function. In addition, the animals developed mild to moderate PE in hypoxia. α-Adrenergic mechanisms are involved in the formation of this hypoxic PE. Our data strongly indicate that the physiological degree of hypoxic sympathetic activation has to be finely balanced. Both too strong and too weak stimulation of the adrenergic system may have detrimental cardiopulmonary consequences. This may have important implications for tourists rapidly ascending to high altitude as well as for patients suffering from hypoxia and hypoxemia.

## Data Availability

The raw data supporting the conclusions of this manuscript will be made available by the authors, without undue reservation, to any qualified researcher.

## Ethics Statement

The experiments were conducted in accordance with the Guide for the Care and Use of Laboratory Animals published by the National Institutes of Health and with the “European Convention for the Protection of Vertebrate Animals used for Experimental and other Scientific Purposes” (Council of Europe, 1986). All animal protocols were approved by the Federal State Agency.

## Author Contributions

BR, CB, and PG contributed to the conception and design of the study. BR, CB, and PA performed the animal experiments, hemodynamic measurements, and bronchoalveolar lavage. PG prepared the histological slices and stainings. CB and PA performed the clinical assessments, evaluated the hemodynamic data, and determined the lung W/D ratios. AS and CB measured the serum catecholamine concentrations. KS and PG evaluated the histological slices. BR, CB and PG performed the statistics. BR wrote the first draft of the manuscript. CB and PG contributed to sections of the manuscript. All authors contributed to the final version of the manuscript and read and approved the submitted version.

## Conflict of Interest Statement

The authors declare that the research was conducted in the absence of any commercial or financial relationships that could be construed as a potential conflict of interest.
